# Analysis of the failure state of the Cenozoic clay layer in thin bedrock coal seam mining: A case study of Sanyuan coal mine

**DOI:** 10.1371/journal.pone.0337462

**Published:** 2026-03-06

**Authors:** Guangming Wu, Mingbin Wang, Lei Wang

**Affiliations:** School of Hydraulic and Civil Engineering, Ludong University, Yantai, Shandong, China; Ministry of Education, MOROCCO

## Abstract

A mechanical analysis model was constructed for the clay layer. Using a combined approach of theoretical analysis and numerical simulation, the failure state of this layer during coal seam extraction under thin bedrock conditions was investigated. The findings offer insights for evaluating the water-blocking performance of the Cenozoic clay layer and for preventing water inrush disasters in similar geological settings. The key findings are as follows: (1) The most critical factor governing clay layer failure is the amount of bedrock subsidence following collapse. As the subsidence of the fractured bedrock increases, the suspended section of the overlying clay layer extends, elevating its possibility to shear or tensile failure. (2) Variations in bedrock thickness markedly influence the subsidence of both the bedrock and the clay layer. Consequently, bedrock thickness is a primary determinant of the degree of damage sustained by the clay layer. (3) The minimum bedrock thickness required to prevent failure of the basal clay layer within the Cenozoic overburden ranges from 33 m to 38 m. If the bedrock thickness falls below 33 m, the clay layers will be damaged after coal seam mining, leading to a loss of their water-blocking capacity.

## 1 Introduction

With the escalating intensity of coal mining operations in China, a range of environmental challenges have surfaced and are growing ever more critical. Among these, water resource scarcity and surface deterioration stand out as particularly pronounced issues, especially in the mid-western parts of China. These regions boast substantial amounts of latent groundwater resources embedded within the Cenozoic overburden. Consequently, the issue of water resource depletion caused by coal seam extraction is notably acute [1–3]. To address this, researchers have proposed groundbreaking ideas and strategies, notably “green coal mining” [4–6] and “co-existence mining of coal and water” [7,8]. These approaches provide novel perspectives for protecting water resources during coal mining operations.

During the extraction of coal seams under thin bedrock conditions, damage to the Cenozoic aquifer is particularly pronounced. The clay layers in the Cenozoic overburden serve as important aquicludes during the mining of thin bedrock coal seams. Numerous studies have examined the failure mechanisms of these clay layers and their water-blocking effectiveness during coal extraction beneath Cenozoic aquifers. For example, the notion of the risk coefficient for water inrush from loose aquifers has been introduced, and it is widely recognized that the clay layer at the bottom of the Cenozoic overburden can serves as an indispensable component of the mining protective barrier [9–11]. Moreover, research indicates that bedrock thickness significantly influences the degree of damage sustained by the Cenozoic clay layer [12,13]. Concurrently, methods such as multifractal analysis have proven effective in predicting mining-induced rock damage [14–16]. All these findings provide vital insights for the safe mining of coal seams under thin bedrock conditions.

When conducting coal seam mining under thin bedrock circumstances, bedrock failure exhibits unique dynamic pressure features and step-like subsidence patterns, setting it apart from the failure behavior of bedrock with normal thickness [10,17–20]. Reduced bedrock thickness exacerbates mining-induced damage in the overlying strata, promoting the rapid upward propagation of water-conducting fractures. This behavior has been confirmed through both numerical simulations and similarity simulations [21–25]. Once water-conducting fractures penetrate through the bedrock, the clay layer located at the bottom of the Cenozoic overburden takes on a crucial function as a water blocking formation. it serves as the primary barrier against water from loose aquifers rushes into the working face. Meanwhile, factors such as the clay layer’s properties (physical properties, distribution, and failure characteristics) and the chosen grouting repair method significantly affect the ultimate water-blocking performance [26–29]. Currently, the failure characteristics of the overlying soil layer and the water blocking mechanism of this overlying soil layer during coal seam mining under thin bedrock conditions represent pressing research issues that demand thorough investigation. This paper, taking into account the geological conditions of thin bedrock in the No.3 mining area of Sanyuan coal mine, adopts theoretical analysis and numerical simulation approaches to analyze the failure state of clay layer. The findings aim to provide a valuable reference for other mining regions with similar geological conditions.

## 2 General situation of engineering geology

**Fig 1:**
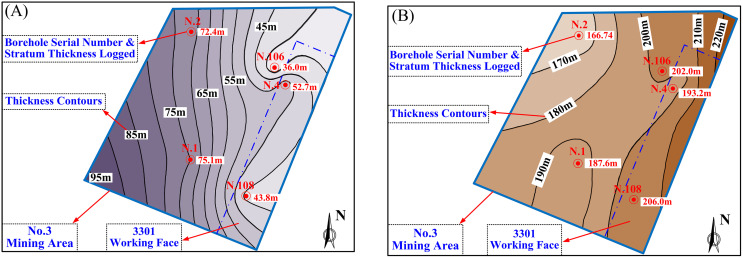
Contour map of stratum thickness. (A) Bedrock thickness. (B) Cenozoic overburden thickness.

The Sanyuan coal mine is situated in the Changzhi Basin, located in the southeastern part of the Qinshui Coalfield. The entire area is overlain by Cenozoic loose deposits, with a general stratum dip angle below 8^0^ degrees. According to data from ground-exploration boreholes, the basic geological conditions of the No.3 mining area are presented as follows. The thickness of the coal seam ranges from 6.7 meters to 7.5 meters. The bedrock thickness varies from 36.0 meters to 75.1 meters (as illustrated in Fig 1A), and the thickness of the Cenozoic overlying layers on top of the bedrock is between 166.7 meters and 206.6 meters (as shown in Fig 1B). Consequently, coal seam extraction in this area occurs under typical geological conditions characterized by thin bedrock (where the bedrock thickness is less than the height of the water flowing fractured zone) and thick overburden [30]. Mining under such thin bedrock geological conditions frequently triggers water inrush disasters from Cenozoic loose aquifers, posing serious risks to mining enterprises (as shown in Table 1). Therefore, ensuring the safe extraction of coal resources in such geological settings represents an important issue that requires heightened attention.

**Table 1:** Statistics of water inrush disasters during coal mining under thin bedrock conditions in China.

Mining enterprise	Time of disaster occurrence	The impact of disaster
Ciyaowan coal mine	1990.04.20	The basic construction project of the coal mine was forced to stop.
Daliuta coal mine	1993.03.24	Coal mining work forced to stop.
Shangwan coal mine	2001.05.31	The mine transport roadway was submerged by water influx.
Henghe coal mine	2001.10.11	Coal mining work forced to stop.
Shigetai coal mine	2008.11.09	Large scale subsidence of the earth’s surface, the mine transport roadway was submerged by water influx.
Halagou coal mine	2010.07.28	Large scale subsidence of the earth’s surface, coal mining work forced to stop.
Renlou coal mine	2012.08.12	Coal mining work forced to stop.
Longde coal mine	2012.11.17	Large scale subsidence of the earth’s surface, coal mining work forced to stop.
Zhaogu coal mine	2019.04.24	Coal mining work forced to stop.

**Fig 2:**
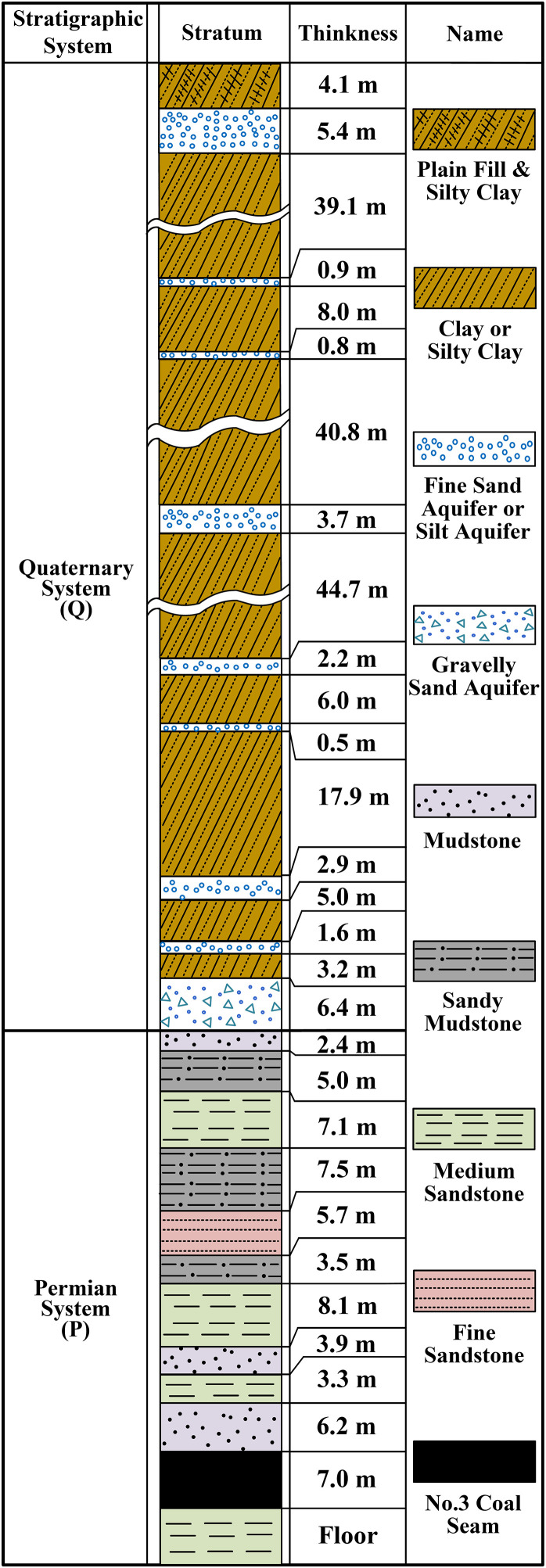
The stratigraphic situation revealed by N.4 borehole.

Within the No.3 mining area, the Cenozoic overburden mainly consists of reddish-brown and yellowish-brown clay or silty clay. Moreover, this region is home to several loose aquifers that hold a moderate volume of water. The clay layers and loose aquifers are arranged in a stratified pattern, and their distribution features are clearly depicted in the borehole column diagram (as presented in Fig 2). Analysis of borehole samples reveals the following key characteristics of the Cenozoic overburden soils. The strata are predominantly composed of clay, with a plasticity index (I_P_) exceeding 17. A subordinate portion consists of silty clay, having an I_P_ greater than 10 but ≤17. For most of the clay and silty clay situated at relatively shallow burial depths, the liquidity index (I_L_) lies between values greater than 0 and less than or equal to 0.75. This indicates that they are in a state ranging from plastic to hard-plastic. Conversely, a majority of the clay and silty clay located at deeper burial depths have an I_L_ less than or equal to 0, which means they are in a hard state. Clay with these properties demonstrates good engineering behavior, rendering it a suitable structure to serve as an effective water blocking layer before it undergoes failure [26,27].

## 3 Theoretical analysis of clay layer failure

### 3.1 Deformation and force analysis of clay layer

**Fig 3:**
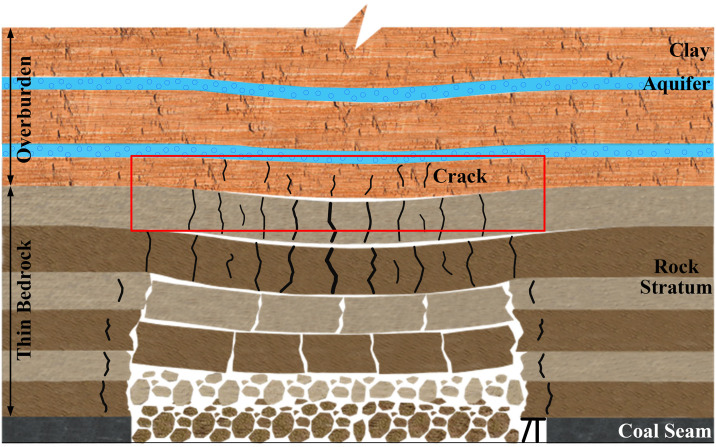
Collaborative deformation and failure of clay layer and bedrock.

With the ongoing extraction of coal seams and the subsequent failure of the thin bedrock, the overlying clay layer atop the thin bedrock is bound to experience deformation or even failure in conjunction with the bedrock’s deformation (as depicted in Fig 3). As a result, the failure of the clay layer is intimately linked to the deformation of the bedrock.

**Fig 4:**
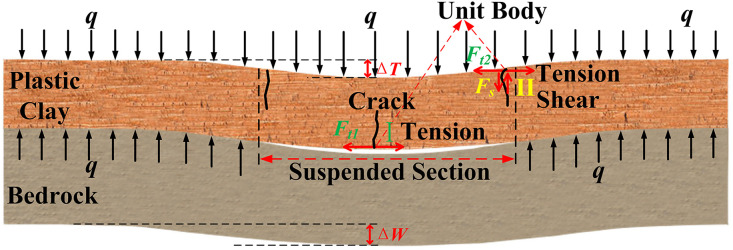
Deformation and force diagram of clay layer.

The deformation and force-related analysis of the clay layer are illustrated in Fig 4. As previously stated, clay demonstrates excellent plasticity and can withstand a certain degree of deformation without incurring damage. The following describes its deformation and load-bearing characteristics. Firstly, the clay layer and the underlying bedrock subside and deform in a synchronized manner. As the bedrock undergoes rapid collapse, a specific gap emerges between the bedrock and the clay layer. The magnitude of this gap is equivalent to the difference between the subsidence of the bedrock (denoted as ΔW) and the subsidence of the clay layer (denoted as ΔT). Secondly, the bedrock, which serves as the supporting structure for the overlying soil layers, bears the majority of the overlying load q. A minor portion of the overlying load q is supported by the clay layer in the suspended section. Thirdly, the clay layer is subjected to both shear force and tension force in the boundary area of the suspended section (area II in Fig 4). Meanwhile, in the middle region (area I in Fig 4) of the suspended section, it is only under tension force. Fourthly, the subsidence values of the clay layer (ΔT) and the bedrock (ΔW) are crucial factors in determining the failure of the clay layer. This is because as ΔT and ΔW increase, both the shear deformation and tension deformation of the clay layer become more pronounced, leading to an increase in the shear and tension forces.

### 3.2 Analysis of the failure state of area I

**Fig 5:**
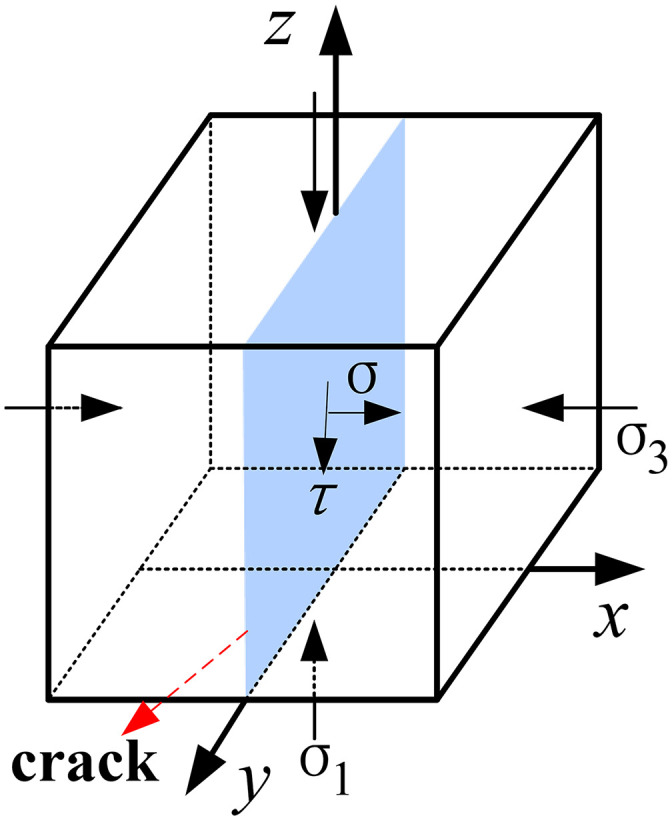
Stress conditions of the unit body.


The x-axis represents the direction in which coal seam mining progresses, the y-axis signifies the widthwise direction of the working face, and the z-axis indicates the vertical direction. Let y = 0 be defined as the central point of the working face width. As a result, the deformation and forces exhibit symmetry with respect to the cross-section that is perpendicular to y = 0. If a unit body (as illustrated in Fig 5) is extracted from the crack area I for stress analysis, the following conditions exist:

$$\sigma_{\text{3}}={-F}_{t\text{1}}\left(C_{p},\Delta T\right),\;{\;\sigma }_{\text{1}}=\text{0}$$ (1)

In equation (1), $F_{t\text{1}}$ denotes the tension force acting on the clay layer. This tension force is a function that depends on the inherent properties of the clay, denoted as $C_{p}$, and the magnitude of subsidence of the clay layer, represented by ΔT.

Based on the Coulomb-Navier criterion, the formula for determining tensile failure at the crack is given by equation (2).


$$\left|\sigma_{\text{3}}\right|\geq \frac{\text{2}C}{\sqrt{\text{1}+f^{\text{2}}}+f}$$ (2)

In equation (2), f stands for the internal friction coefficient of the clay, where f=tanφ. Here, φ represents the internal friction angle of the clay, and c denotes the cohesion.

By applying equations (1) and (2), we can deduce that, in accordance with the failure criterion outlined in equation (3), tensile failure will take place in the clay within area I.


$$F_{t\text{1}}\left(C_{p},\Delta T\right)\geq \frac{\text{2}C}{\sqrt{\text{1}+f^{\text{2}}}+f}$$ (3)

### 3.3 Analysis of the failure state of area II

When a unit body (as depicted in Fig 5) is extracted from the crack area II for the purpose of stress analysis, the following conditions exist:


$$\sigma_{\text{1}}=q_{c}\left(B_{p},\Delta W\right)=q-q_{b}\left(B_{p},-\Delta W\right),\;{\;\sigma }_{\text{3}}={-F}_{t\text{2}}\left(C_{p},\Delta T\right)$$ (4)

In equation (4), $q_{c}$ represents the portion of the total overlying load q that is supported by the clay layer. $q_{b}$ represents the portion of the total overlying load q that is supported by the bedrock. The value of $q_{c}$ is a function dependent on the lithological characteristics of the bedrock, denoted as $B_{p}$, and the magnitude of the bedrock’s subsidence, indicated by ΔW. As the bedrock’s subsidence amount increases, the suspended portion of the clay layer becomes longer, leading to an increase in the load carried by the clay layer. The value of $q_{b}$ is a function dependent on the lithological characteristics of the bedrock and the magnitude of the bedrock’s subsidence. As the bedrock’s subsidence amount increases, the suspended portion of the clay layer becomes longer, leading to a decrease in the load carried by the bedrock. $F_{t\text{2}}$ denotes the tension force acting on the clay layer. This tension force is a function that depends on the inherent properties of the clay and the magnitude of subsidence of the clay layer.

Based on the Coulomb-Navier criterion, the formula used to determine tensile failure at the crack area II is presented as equation (2). The formula used to determine shear failure at the crack area II is presented as equation (5)


$$\sigma_{\text{1}}\left(\sqrt{\text{1}+f^{\text{2}}}-f\right)-\sigma_{\text{3}}\left(\sqrt{\text{1}+f^{\text{2}}}+f\right)\geq \text{2}C$$ (5)

By applying equations (4) and (2), we can deduce that, in accordance with the failure criterion outlined in equation (6), tensile failure will take place in the clay within area II.


$$F_{t\text{2}}\left(C_{p},\Delta T\right)\geq \frac{\text{2}C}{\sqrt{\text{1}+f^{\text{2}}}+f}$$ (6)

By drawing upon equations (4) and (5), we can infer that, in line with the failure criterion set forth in equation (7), shear failure will manifest in the clay within area II.


$$q_{c}\left(B_{p},\Delta W\right)\left(\sqrt{\text{1}+f^{\text{2}}}-f\right)+F_{t\text{2}}\left(C_{p},\Delta T\right)\left(\sqrt{\text{1}+f^{\text{2}}}+f\right)\geq \text{2}C$$ (7)

According to formulas (3), (6), and (7), in addition to the intrinsic properties of the clay layer and the bedrock, the factors that exert a substantial influence on the shear or tensile failure of the clay layer encompass the subsidence amount of the clay layer and that of the bedrock. In the No.3 mining area of Sanyuan coal mine, parameters like the properties of the bedrock and the clay are already determined. However, the variable element is the thickness of the bedrock, as illustrated in Fig 1. In other words, within this specific mining area, the principal factor that impacts the subsidence of both the clay layers and the bedrock is the variation in bedrock thickness. Consequently, a numerical simulation approach is employed to examine the effects of bedrock thickness variations on the failure behavior of the clay layer.

## 4 Numerical simulation analysis of clay layer failure

### 4.1 Numerical model overview

A numerical model was established in accordance with the geological conditions revealed by N.4 borehole, as depicted in Fig 2. Keeping the thickness of the Cenozoic overburden constant at 186 m, five different schemes were designed by successively reducing the bedrock thickness to simulate its variations. More precisely, the bedrock thickness was set to 53 m, 48 m, 43 m, 38 m, and 33 m in the respective schemes.

**Fig 6:**
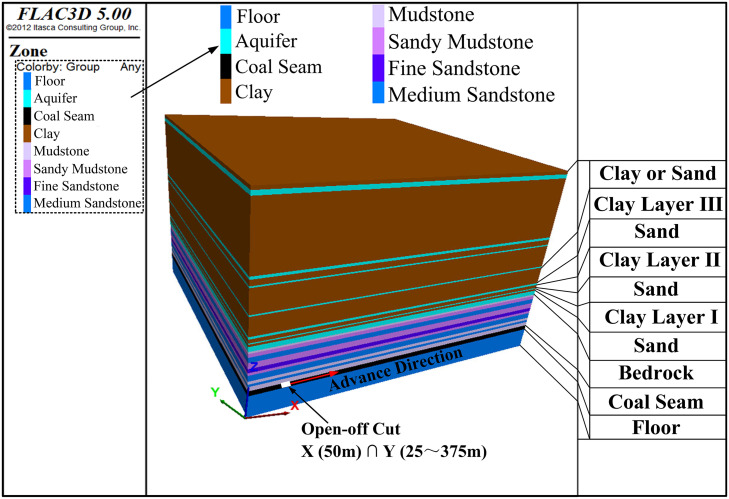
Numerical simulation model.

Using the scheme with a bedrock thickness of 53 m as an example, the following provides a concise overview of the approach used to construct a numerical model. After the integration of certain strata, the resulting model measures 400 m in length, 400 m in width, and 276 m in height, as illustrated in Fig 6. The model was constructed using brick unit, comprising a total of 825600 units. Geotechnical materials were represented by the Mohr-Coulomb elastoplastic constitutive model, which is widely used for common rock and soil materials. Numerical simulations were conducted in FLAC3D, which employs a fast Lagrangian analysis of continua approach that does not permit unit separation. The physical and mechanical properties of each rock and clay stratum are detailed in Table 2. The mechanical parameters presented in Table 2 were derived from two primary sources: laboratory testing and previously published literature. Laboratory tests provided direct measurements of cohesion, internal friction angle, and density for the clay layers, as well as tensile strength and density for the rock units. Parameters that could not be experimentally determined due to equipment or sample limitations were adopted from established references [10,30]. These literature-sourced values were obtained from adjacent coal mines within the same mining area and under comparable geological conditions, ensuring their applicability to the present study.

**Table 2:** Physical and mechanical parameters of the model.

Lithology	Modulus of elasticity (GPa)	Poisson’s ratio	Cohesion (MPa)	Tensile strength (MPa)	Internal friction (^0^)	Density (kg/m^-3^)
Sand	10.000kPa	----	200.000Pa	200.000Pa	20.000	1690.000
Clay	0.021	0.350	0.162	0.195	28.600	1990.000
Fine Sandstone	38.000	0.250	5.910	1.980	24.400	2744.000
Medium Sandstone	35.000	0.250	5.200	1.090	30.500	2672.000
Sandy Mudstone	16.300	0.260	3.450	0.590	29.000	2548.000
Mudstone	12.100	0.280	2.300	0.470	20.900	2640.000
Coal Seam	5.000	0.230	2.030	0.310	43.000	1600.000
Floor	35.000	0.250	5.200	1.520	30.500	2672.000

In the model, the X-axis is oriented parallel to the direction of working face advance. It spans 400 m in length, with a 50 m boundary zone allocated on either side. The Y-axis corresponds to the width direction of the working face. Since the actual width of the 3301 working face is 350 m, the Y-axis length of the model is set to 400 m, also includes 25 m boundary zone on both ends. The Z-axis is oriented vertically, reaching a height of 276 m. This height consists of the following strata from bottom to top: a 30 m floor, a 7 m coal seam, a 53 m bedrock stratum, and a 186 m overburden layer.

Regarding the model boundary conditions, the bottom (Z = 0 m) is established as a displacement constraint, preventing any displacement in the normal direction. The top (Z = 276 m) serves as an unrestricted boundary, permitting free displacement. The front (Y = 0 m), rear (Y = 400 m), left (X = 0 m), and right (X = 400 m) of the model are configured as displacement boundaries that solely restrict displacement in the normal direction. For the excavation simulation, each step advances the working face by 10 m. The excavation commences at an open-off cut situated 50 m from the model’s left boundary. Following this, the excavation progresses incrementally towards the right, following the X-direction. Overall, this simulation encompasses 30 excavation steps.

### 4.2 The influence of bedrock thickness on clay layer failure

**Fig 7:**
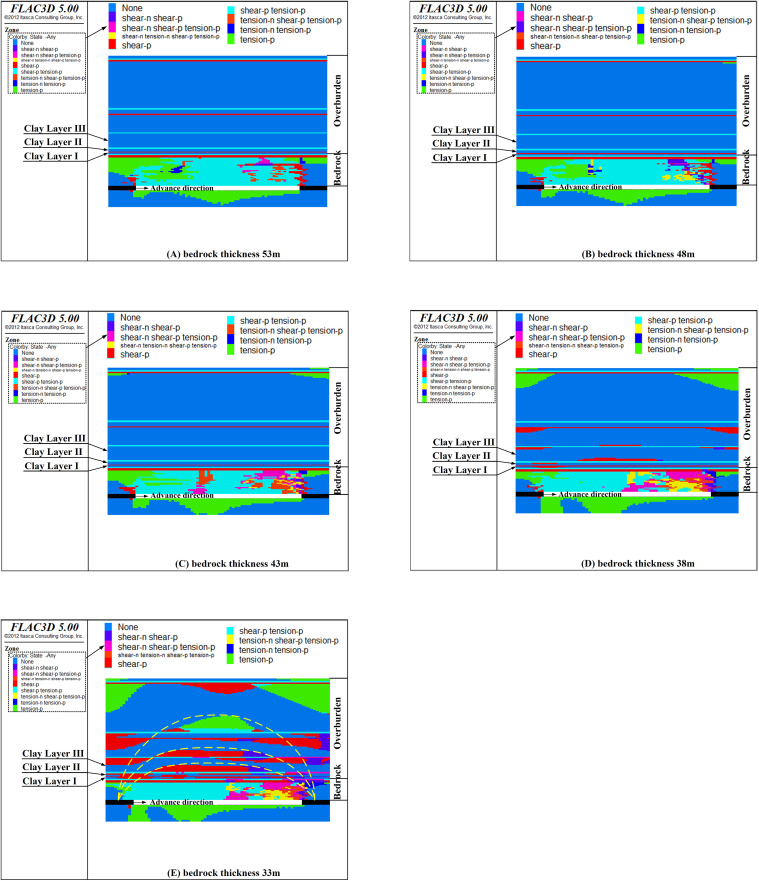
The failure state of clay layers with different bedrock thicknesses (Y = 200 m section).

Upon the completion of all 30 excavation phases, the failure conditions of the clay layer situated above bedrock of different thicknesses are illustrated in Fig 7.

As shown in Fig 7, when the bedrock thickness exceeds 38 m, mining-induced fractures penetrate the bedrock but are arrested at the basal clay layer of the Cenozoic overburden, as demonstrated in Fig 7A-D. The clay layer at the bottom of the Cenozoic overburden acts as a barrier, impeding the propagation of fractures. However, when the bedrock thickness decreases to 33 m, interconnected failure zones begin to develop in clay layers I, II, and III, forming an approximate arch-shaped configuration (as shown in Fig 7E).At this stage, the damaged area extends to the middle and upper parts of the Cenozoic overburden, forming a connected damaged area between the Cenozoic aquifer and the mining face. Consequently, a minimum bedrock thickness of 33 m to 38 m is required to prevent damage to the Cenozoic clay layers.

### 4.3 The influence of advance distance on clay layer failure

**Fig 8:**
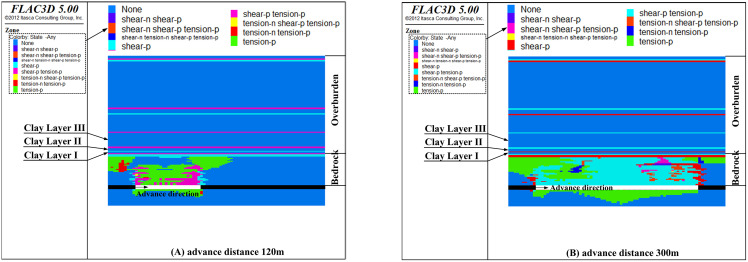
The failure state of clay layers with bedrock thickness 53 m (Y = 200 m section).

**Fig 9:**
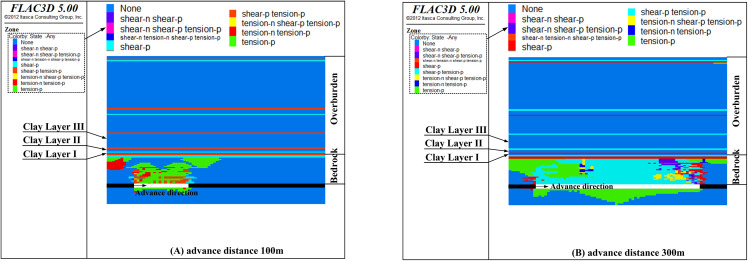
The failure state of clay layers with bedrock thickness 48 m (Y = 200 m section).

**Fig 10:**
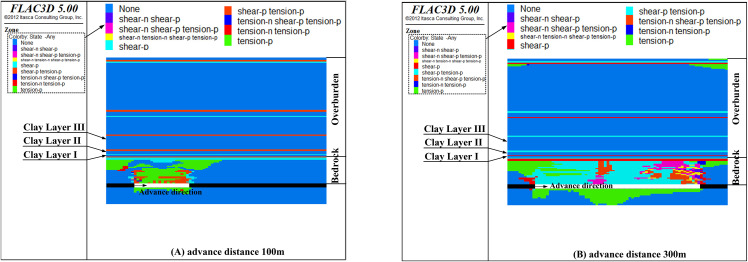
The failure state of clay layers with bedrock thickness 43 m (Y = 200 m section).

**Fig 11:**
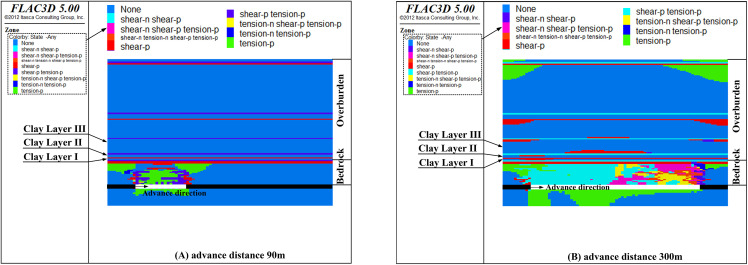
The failure state of clay layers with bedrock thickness 38 m (Y = 200 m section).

Figs 8, 9, 10, 11 show the failure characteristics of clay layers at different advance distances when the bedrock thickness is 53m, 48m, 43m, and 38m, respectively.

As shown in Fig 8, 9, 10, 11, when the bedrock thickness exceeds 38 m, the rock strata within the bedrock become damaged but the damaged area will stop propagating upwards. This indicates that the basal clay layer of the overburden effectively inhibits fracture propagation. Meanwhile, as the bedrock thickness decreases, the advance distance required for complete failure of the bedrock strata also decreases. Specifically, complete failure of the bedrock strata occurs at an advance distance of 120 m for a bedrock thickness of 53 m, at 100 m for thicknesses of 48 m and 43 m, and at 90 m for a thickness of 38 m.

**Fig 12:**
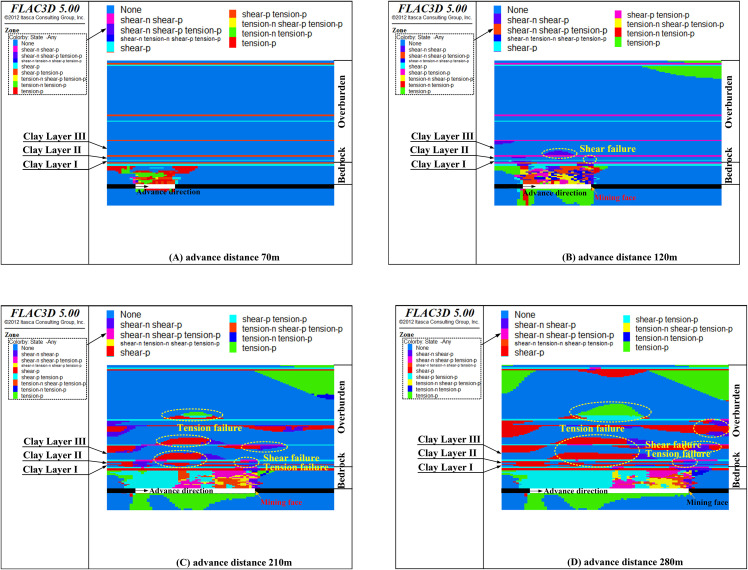
The failure state of clay layers with bedrock thickness 33 m (Y = 200 m section).

Fig 12 presents the failure features of the clay layer at different advance distances when the bedrock thickness is reduced to 33 m.

As illustrated in Fig 12, at an advance distance of 70 m, all rock strata within the bedrock are damaged, but the damage does not propagate beyond it (as depicted in Fig 12A). The clay layer at the bottom of the Cenozoic overburden exerts a retarding influence on the development of cracks. Shear failure in this clay layer does not begin until the advance distance exceeds 120 m (as depicted in Fig 12B). When the advance distance increases to 210 m, the damaged zone extends into the middle section of the Cenozoic overburden (as shown in Fig 12C). As the advance distance further increases to 280 m, the damaged zone expands upward into the upper section of the Cenozoic overburden (as shown in Fig 12D). Overall, the clay layer above the mining face undergoes both shear and tensile failure, whereas the clay layer above the center of the goaf experiences primarily tensile failure. The distribution characteristics of these damaged areas are basically consistent with theoretical analysis.

## 5 Conclusion

This study is based on the engineering conditions of the No.3 mining area in the Sanyuan coal mine. Through theoretical analysis and numerical simulation, it examines the failure state of the clay layer within the Cenozoic overburden during the extraction of a thin-bedrock coal seam. The main conclusions are as follows.

(1) The failure of the clay layer is governed not only by its intrinsic properties but also by the post-collapse subsidence of the bedrock and the subsidence of the clay layer itself. A greater disparity in subsidence between the clay layer and the bedrock results in a longer suspended portion of the clay layer. Consequently, the clay layer becomes more susceptible to shear or tensile failure at the edge or the central region of the suspended segment.
(2) When the properties of the clay and bedrock are ascertainable via geological drilling, variations in bedrock thickness become a key factor controlling the subsidence of both the bedrock and the clay layer. In other words, bedrock thickness substantially influences clay layer failure. Given a Cenozoic overburden thickness of 186 m and a coal seam thickness of 7 m, the minimum bedrock thickness required to prevent failure of the basal clay layer in the Cenozoic overburden ranges from 33 m to 38 m.
(3) When the bedrock thickness exceeds 33 m, the failure zone penetrates the bedrock strata but is arrested at the basal clay layer of the Cenozoic overburden. This indicates that the clay layer effectively inhibits fracture propagation. In contrast, when the bedrock thickness is less than 33 m, the damaged zone gradually extends from the basal clay layer upward into the middle and upper clay layers of the Cenozoic overburden as the advance distance increases. In general, the clay layer above the center of the goaf undergoes tensile failure, whereas the clay layer above the mining face experiences both shear and tensile failure.

## Supporting information

S1 Data Relevant Data. (RAR)
